# Excitability of Motor Cortices as a Function of Emotional Sounds

**DOI:** 10.1371/journal.pone.0063060

**Published:** 2013-05-07

**Authors:** Naeem Komeilipoor, Fabio Pizzolato, Andreas Daffertshofer, Paola Cesari

**Affiliations:** 1 Department of Neurological, Neuropsychological, Morphological and Movement Sciences, University of Verona, Verona, Italy; 2 MOVE Research Institute, Vrije Universiteit University Amsterdam, Amsterdam, The Netherlands; University of Montreal, Canada

## Abstract

We used transcranial magnetic stimulation (TMS) to clarify how non-verbal emotionally-characterized sounds modulate the excitability of the corticospinal motor tract (CST). While subjects were listening to sounds (monaurally and binaurally), single TMS pulses were delivered to either left or right primary motor cortex (M1), and electromyographic activities were recorded from the contralateral abductor pollicis brevis muscle. We found a significant increase in CST excitability in response to unpleasant as compared to neutral sounds. The increased excitability was lateralized as a function of stimulus valence: Unpleasant stimuli resulted in a significantly higher facilitation of motor potentials evoked in the left hemisphere, while pleasant stimuli yielded a greater CST excitability in the right one. Furthermore, TMS induced higher motor evoked potentials when listening to unpleasant sounds with the left than with the right ear. Taken together, our findings provide compelling evidence for an asymmetric modulation of CST excitability as a function of emotional sounds along with ear laterality.

## Introduction

The presence of emotion can be considered a vital prerequisite for proper daily functioning as it helps qualifying information and, by this, (fine) tunes behavioral responses. For nearly three decades, the field of ‘affective neuroscience’ has attracted widespread interest with the overarching aim to decipher the code of emotions. In the human brain, the two hemispheres have certainly distinct roles in the de- and encoding procedures, but how this is explicitly instantiated is still a matter of debate. In this study, we sought to clarify to what degree non-verbal emotionally characterized sounds presented separately to the left and right ear yield differential and possibly lateralized excitability of the corticospinal motor tract (CST).

The close link between action readiness and emotion has been manifested through different experimental approaches. Behavioral studies have shown that selective biases exists in motor responses to emotional valence of visual stimuli [1]–[4] in terms of reduced reaction time [5], [6], increased amplitude of force production [6], [7], and modulated postural adjustments [8]–[10]. Neuroimaging studies revealed that viewing fearful body expressions is accompanied by enhanced activity in motor areas, suggesting a close link between emotion and action preparation [11], [12]. Transcranial magnetic stimulation (TMS) revealed a non-trivial relationship between action preparedness and emotional processing by means of increased corticospinal motor excitability during emotional experiences [13]–[21]. Despite the relatively large number of studies investigating motor responses to emotive aspects of visual stimuli [14], [15], [17]–[19], there are surprisingly few about CST excitability as a function of auditory processing of sounds carrying emotional contents [13], [21]. Using TMS, Baumgartner and co-workers [13] found that simultaneous presentation of pictures and pieces of music with congruent emotional content led to larger amplitudes of motor evoked potentials (MEPs) than in the cases in which the stimuli were presented separately. More recently, Baumert and co-workers [21] reported that the presentation of spoken scenarios describing negative events yielded an increase in the corticospinal facilitation (increased MEPs), as compared to neutral scenarios. Interestingly, they did not find any modulation of CST excitability in response to positive scenarios. This outcome agrees with previous studies [14], [17] but contrasts others that reported increased MEPs in response to both pleasant and unpleasant as compared to neutral stimuli [13], [15], [18], [19]. These contradictory results may be due to various differences in experimental designs across studies. A reason for these differences could be that in all but one study only a single hemisphere has been stimulated: TMS was applied either over the left [13]–[15], or over the right M1 [19]. The one exception is an old study by Tormos and co-workers [20] demonstrating hemispheric differences in motor facilitation during emotional experiences. They found MEPs elicited over the left hemisphere during imagined sad thoughts to be increased, whereas happy thoughts resulted in significantly larger MEPs when elicited over the right hemisphere.

Current models of emotional asymmetry convincingly sustain the existence of distinct processes for emotional encoding within the two halves of the brain, but, as mentioned, the specific involvement of the two hemispheres is yet unclear. Two major models have been put forward. Central to the first is the idea that the right hemisphere is solely, or at least more, involved in processing emotional information than the left one [2]–[4], [22]–[33]. This idea has been around for a long time, and it continues to receive experimental support. For instance, several studies have shown that patients with lesions in the right hemisphere have increased difficulties perceiving both negative and positive emotions [30]–[33] or just negative ones [34]–[36]. In a recent review on unconscious emotional processing, Gainotto suggested the critical role of right hemisphere in involuntary generation of all emotions [37]. However, Sackeim and co-workers [38] reported that damage to the left hemisphere led to depressive symptoms, while to the right one caused pathological laughing behavior. This may suggest a differential activation pattern in both hemispheres, which forms the basis of the second model.

The second model builds on two hypotheses: (i) the *valence hypothesis* and (ii) the *“motivational direction (approach-withdrawal) hypothesis”*. The first one builds on the idea that the left hemisphere is specialized for processing positive emotions, whereas negative emotions are lateralized toward the right hemisphere [39]–[47]. The second hypothesis posits that hemispheric asymmetry of emotional processing is particularly relevant for approach-withdrawal behaviors (hence the notion of “motivational approach-withdrawal model”). That is, the left hemisphere is lateralized for approach- and the right one for avoidance-related emotions [48].

Despite the large number of findings supporting the valence and motivational models, this might not be the end of the story. In fact, there are many studies suggesting emotion-related activation patterns in the brain cannot be merely appointed to either the valence or the motivational hypothesis [49]–[54]. Damasio and co-workers showed that brain activities for emotions are better represented by dynamic distributed neural maps, which suggests a no clear-cut preferences between hemispheres as far as emotional processing is concerned [54]. Wager and co-workers [55] performed a meta-analysis regarding the results obtained from several neuroimaging studies that evaluated brain asymmetry on emotional processing. They found no hemispheric differences when each hemisphere was analyzed as a whole, whereas, as soon as smaller brain regions were studied, brain asymmetry was identified. Wager and co-workers hence concluded that the lateralization of emotional activity is region-specific, which led us to restrict our study to left and right M1s. In fact, to date most imaging studies on emotional response have focused on activities in prefrontal cortex and amygdala. Much less attention has been devoted to M1, although it may be one of the most important brain regions in processing emotional salience by virtue of action readiness [13]–[21]. We, therefore, tested the presence of an asymmetrical modulation in the motor cortex in response to non-verbal emotional sounds.

In terms of ear asymmetry, sounds can be perceived mon- or binaurally, which may have differential effects on left/right motor facilitation. To our best knowledge, this is the first study to test ear laterality in CST excitability. Earlier behavioral studies on ear asymmetry employed dichotic listening tests in order to assess ear superiority in processing different auditory information. Dichotic listening method is a technique consisting of a simultaneous presentation of two different stimuli, one to each ear, to create competition in processing the stimuli between the two ears. Early dichotic listening research evidenced right-ear advantage for processing verbal information [56], [57]. Kimura considered this to be the consequence of a strong connection between ears and contralateral hemispheres [57]–[58]. Accordingly, verbal stimuli presented to the right ear travel preferably to the left hemisphere, which contains the language processing areas, and hence the right-ear advantage appears. As regards ear laterality in terms of emotion, many studies reported left ear advantage in processing both verbal and non-verbal emotional sounds regardless of their valence (positive-negative) [59]–[64]. This has been considered to support the right hemisphere hypothesis. It has also been shown that ear laterality applies not only to the accuracy of performance, but also to the speed of the response time [65]–[68]. Interestingly, in a study by Gagnon and Peretz, [66] subjects were presented with monaural tonal and atonal melodies and were instructed to evaluate the level of pleasantness while response time was measured. They found faster responses for tonal melodies presented to the right ear, whereas atonal melodies were detected more quickly when presented to the left ear. Kallman [67] reported shorter reaction time in response to verbal stimuli presented to the right ear and in response to non-verbal sounds presented to the left one. Similarly, in a study by Kallman and Corballis [68] subjects were able to recognize pieces of music faster with the left-ear compared to the right one. Overall, the dissimilarities in performance between ears are thought to indicate the dominance of the contralateral hemisphere in processing auditory stimuli. However, none of these aforelisted experiments included a direct assessment of brain activity. It is also not clear how left and right motor cortices respond differentially to non-verbal emotional stimuli delivered to different ears. We employed TMS to clarify whether non-verbal emotionally-characterized sounds delivered to either ear separately or to both ears modulate MEPs differently.

Our overall aim was to test whether (i) emotional processing of non-verbal auditory stimuli would lead to increased CST excitability. We hypothesized that (ii) this modulation of CST excitability to be lateralized in response to the valence of the stimuli, and that (iii) delivering the sounds to the left ear, right ear, or both ears may yield lateralization in motor facilitation. We expected that emotional sounds would facilitate CST excitability similar to affective visual stimuli, conceivably in order to tune the appropriate reaction in the presence of different emotional stimuli. To our knowledge, the only study that measured the CST excitability in both hemispheres was the one performed by Tormos and co-workers [20]. In line with their result, we expected unpleasant sounds to result in a selective facilitation of the MEPs elicited over left-hemisphere and pleasant sounds to yield a higher activation following the stimulation of right hemisphere. In view of earlier studies addressing the superiority of the left ear for the perception of non-verbal emotional sounds, we finally expected that listening to emotional stimuli (specifically unpleasant sounds) with the left ear leads to a higher CST excitability as compared to neutral sounds.

## Methods

### Ethics Statement

The experimental protocol was approved by the members of the Ethics Committee of the Department of Neurological, Neuropsychological, Morphological and Movement Sciences of the University of Verona (Protocol number 232). All participants provided their written informed consent prior to entering the study, which had been approved by the institutional review board.

### Participants

Thirteen healthy right-handed volunteers as measured by the Edinburgh Handedness Inventory [69] (six females; 25.2±3.8 years) were recruited at University of Verona to participate in the experiment for either extra academic credit or financial equivalent. Before participating in the study, all the participants performed a self-hearing test [70]. One participant was excluded from analysis due to poor task performance and excessive hand movement during the experiment.

### Stimuli

Sound stimuli were selected from the International Affective Digitized Sounds [71]: a set of 111 standardized, emotionally evocative sounds that are characterized along the affective dimensions of valence (ranging from pleasant to unpleasant), arousal (ranging from calm to excited), and dominance (ranging from in control to dominated). Sounds were intended to differ significantly in valence dimension but not in arousal. We chose fifteen different non-verbal sounds, five of which are categorized as unpleasant (explosion, siren, man sobbing, buzzing, dentist drill), five as pleasant (rock and roll music, baby laughing, Bach’s music, ocean and seagulls, babbling brook), and five as neutral (restaurant ambience, walking, heartbeat, clock ticking, toilet flush); see Appendix S1. This categorization was confirmed after analyzing subjective valence and arousal rating scores performed by subjects for all the sounds (Table 1&2). The sound intensities were adjusted using RMS (Root Mean Square) equalization in MATLAB 7.7.0 (Mathworks Inc). In order to prevent the acoustic startle-reflexes/off-responses, the intensity of sound stimuli was gradually increased/decreased within the first/last seconds by using conventional fading-in/out. The maximum peak amplitude for all the sound was set to 0 dB FS (decibels relative to full scale). For all the fifteen sounds, monaural right and left version were created, and then all the sounds (45 stimuli) were converted to 16-bit wav files. All these conversions were realized with GarageBand '11 (Apple, Inc). We used the E-Prime2 software running on a PC with a Windows XP operating system to control the stimulus presentation. The stimuli were presented at a constant (maximum) volume level for all subjects.

**Table 1:** Subjective and Normative Mean Valence Rating Scores on a Scale Ranging From 1 (Very Pleasant) to 9 (Very Unpleasant).

	Valence Category					
	Unpleasant		Neutral		Pleasant	
	M	SE	M	SE	M	SE
Subjective ratings	2.48	0.23	4.80	0.18	7.31	0.25
Normative ratings	3.09	0.17	4.80	0.36	7.30	0.51

**Table 2:** Subjective and Normative Mean Arousal Rating Scores on a Scale Ranging From 1 (Very Calm) to 9 (Very Excited).

	Arousal Category					
	Unpleasant		Neutral		Pleasant	
	M	SE	M	SE	M	SE
Subjective ratings	6.56	0.10	4.27	0.37	6.59	0.29
Normative ratings	6.54	0.21	4.43	0.29	5.11	0.61

### Procedure

TMS induced electromyographic (EMG) activity as well as subjective valence and arousal ratings were collected from all participants.

**Figure 1:**
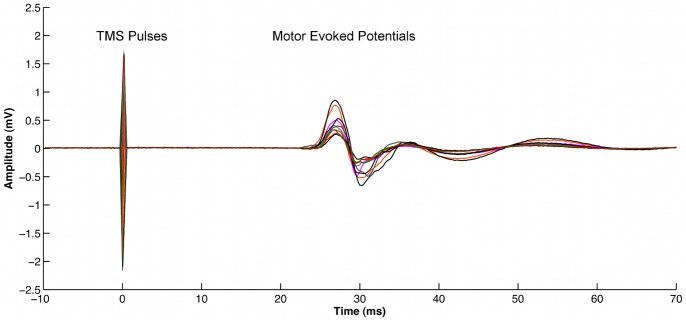
Examples of ten motor evoked potentials (MEPs) recorded in a resting abductor pollicis brevis (APB) muscle during one condition in a single subject. The vertical lines at 0 ms indicates when a single pulse of TMS was fired.

During all sessions, participants wore conventional earphones (Beyer Dynamic DT-770) and were seated in a comfortable chair. Auditory stimuli were presented for the duration of six seconds via earphones binaurally (both ears) or monaurally (right or left ears). Single pulse TMS was delivered to either the right or the left M1 while participant were listening to sounds (ten MEPs during each condition, yielding a total of 180 MEPs; see below and cf. figure 1). The order of stimulation site (right or left) was counterbalanced across subjects with a rest period of five to ten minutes between sessions. TMS pulses were applied at inter-stimulus intervals of ten seconds and delivered randomly at 2, 3 or 4 seconds after the stimulus onset. Participants were instructed to attend to stimuli and report what they heard after the entire sound had been played. The order of stimulation site (left or right) was counterbalanced across participants. After the TMS sessions, ratings of valence (pleasant-unpleasant), and arousal (calm-excited) were collected in separate sessions from all participants. For both TMS and rating sessions, the order of stimulus presentation was randomized according to the hearing condition (right/left) and the valence component of the stimuli (pleasant/unpleasant/neutral).

### Data Acquisition

Focal TMS was applied with a 70-mm figure-of-eight coil that was powered by a Magstim 200 Rapid stimulator (Magstim, Whitland, Dyfed, UK) producing a maximum output of 2T at the coil surface. The TMS coil was placed tangentially on the scalp over the ‘optimal scalp site’ to elicit MEPs in the right (and left) abductor pollicis brevis (APB) muscle. The optimal scalp site was defined as the scalp position and coil orientation where TMS-induced MEPs were stable and maximal in the APB muscles. Prior to data collection, the individual resting motor threshold (RMT) for right (and left) APB muscle was measured by delivering single TMS pulses over the contralateral primary motor cortex. RMT was defined as the minimum intensity needed for eliciting MEPs (usually >50 µV) in at least five out of ten TMS pulses when the muscle is completely relaxed [72]. Single pulse TMS was delivered at 130% of the individual resting motor threshold for all trials. Ninety MEPs were recorded from each subject in each hemisphere. MEPs were recorded using Ag-AgCl cup electrodes (10 mm diameter), which were placed over a belly-tendon montage with an inter-electrode distance of ±3 cm, and the ground electrode was attached to the wrist. The electromyogram (EMG) signals were online band-pass filtered (20–3000 Hz), amplified (Digitimer, Hertfordshire, England), and sampled at a rate of 5 kHz using a CED Micro 1401 (Cambridge Electronic Design, Cambridge, England).

Individual valence and arousal rating of the sounds were collected from each subjects in order to control whether it represents the normative rating of IADS and accordingly to define individual emotional categories. Affective ratings took place after the TMS experiment in two separate sessions. The 45 stimuli used in the TMS session (the fifteen sounds for each of the three hearing conditions: left, right, binaural) were presented randomly via earphones for six seconds. For the valence rating, after the presentation of each sound, a valence scale was shown where ‘1’ indicated very pleasant and ‘9’ indicated very unpleasant. Likewise, for the arousal rating, after the presentation of each sound, an arousal scale was displayed, where ‘1’ indicated very calm and ‘9’ indicated very excited. Subjects had to type their number of preference and press the enter button to hear the next sounds. The order of the two sessions was counterbalanced across subjects.

### Data Analysis

MEPs were analyzed off-line using Spike 2 (version 6, Cambridge Electronic Design). First we confirmed the absence of background EMG activity confounding the MEP analysis by visual inspection of the data. To reduce inter-subject variability, individual MEP amplitudes were transformed to their corresponding z-scores based on individual means and standard deviations over all the stimulation trials in each hemisphere. MEPs two or more standard deviation off a subject’s mean (per hand) were excluded from the analysis. On average, for each subject, four out of ninety MEPs per side were excluded (range: 2 to 18 MEPs). In total of 5% of the data were discarded (1.5% for pleasant 1.5% for unpleasant and 2% for neutral sounds).

### Statistics

The TMS experiment contained three factors: *stimulation site* (right and left hemisphere, or RH and LH, respectively), *emotional valence* (unpleasant, neutral, and pleasant), and *hearing* (monaural right and left and binaural, or RE, LE, and BE, respectively). We addressed this 2×3×3 design with a 3-way ANOVA with repeated measures. Post-hoc comparisons were performed by means of t-tests applying the Bonferroni correction for multiple comparisons when required. Mauchly’s Test of Sphericity indicated that the assumption of sphericity had not been violated.

To assess the subjects’ sound ratings we performed two distinct repeated-measures ANOVAs for valence and arousal scales. We further compared each of the valence categories using a t-test as a post-hoc analysis. The statistical assessments were performed using SPSS version 17.0 (SPSS Inc., Chicago IL). Significance level was always set to p<.050 and only significant results are presented.

## Results

### Motor Evoked Potentials

**Figure 2:**
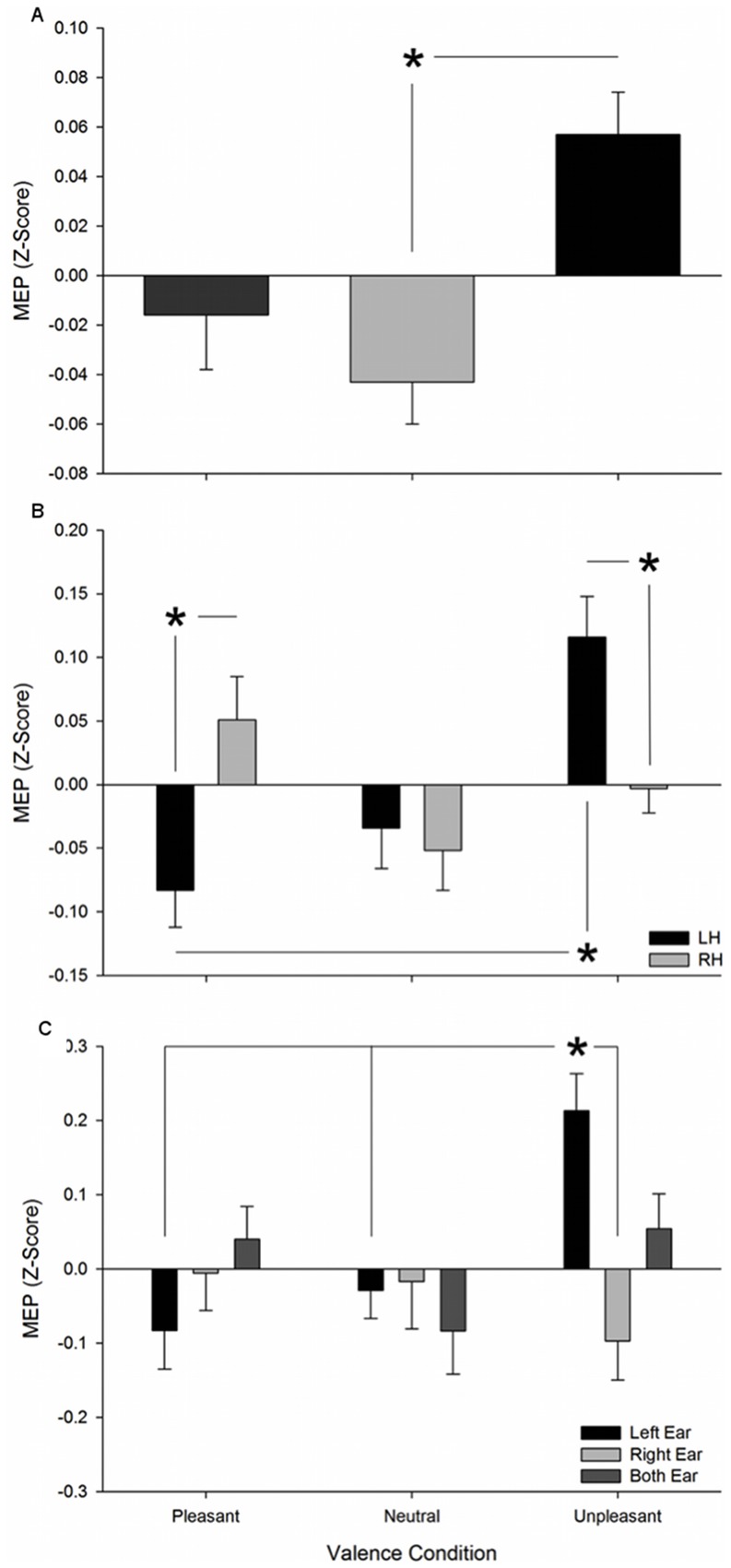
Overall mean (SEM) Motor-Evoked Potential (MEP) amplitude in Z score. (**A**) per valence condition (Pleasant, Neutral, Unpleasant); (**B**) in left and right Motor cortex per valence condition; (**C**) for different Hearing conditions per valence condition. *P<.05.

We found a significant main effect for *emotional valence* (F_2,24_ = 5.047, p = .015) where MEPs were larger when subjects listened to unpleasant as compared to neutral sounds. The interaction between the *stimulation site* and *emotional valence* was also significant (F_2,24_ = 5.037, p = .015). Unpleasant sounds elicited larger MEP amplitudes on the left M1 than on the right one (p = .013). By contrast, pleasant sounds presented selectively larger MEPs on the right hemisphere as compared to the left hemisphere (p = .011). Post-hoc analysis indicated that, when the left M1 was stimulated, unpleasant sounds led to selectively larger excitability of MEPs than pleasant ones (p = .009). The interaction of *hearing* with *emotional valence* was significant (F_4,48_ = 3.452, p = .015). The t-test revealed that listening to unpleasant sounds with the left ear yielded larger MEPs than listening with the right ear (p = .004). On the other hand, MEPs evoked when the sounds delivered to the left ear were significantly larger when unpleasant as compared to neutral (p = .012) and pleasant (p = .004). These results are summarized in figure 2.

### Valence and Arousal Scales

The average valence ratings (± SE) were 7.31±0.25 for the pleasant, 4.8±0.18 for the neutral, and 2.48±0.23 for the unpleasant sounds. Repeated-measures ANOVA confirmed that these valence ratings differed significantly between emotion categories (F_2,24_ = 127.48, p<.0001). Post-hoc paired-samples t-tests indicated that negative, neutral, and positive sounds were evaluated as significantly different from each other (all p<.0001). The mean participant ratings of arousal for the unpleasant, neutral, and pleasant sounds were 6.56±0.10, 4.27±0.37, and 6.59±0.29, respectively. The ratings of arousal differed significantly between emotion categories (F_2,24_ = 25.34, p<.0001). Negative and positive sounds were rated as more arousing than neutral, p<.001 and p<0.0001 respectively; see tables 1 & 2 for an overview.

## Discussion

This study was designed to assess (i) to what degree emotional processing of non-verbal auditory stimuli would modulate the CST excitability, (ii) whether there is an asymmetric modulation of CST excitability in response to the valence of the stimuli, and (iii) if differences in MEPs can be detected while subjects are listening to stimuli with different ears. We here provided direct evidence for a selective motor facilitation as a result of listening to non-verbal emotional sounds. From an evolutionary perspective, one may argue that processing the presence of an emotion is important, if not vital, requisite for survival as it helps to qualify information in the environment for mobilizing the body to perform proper reactions [73], [74]. As such, our findings also contribute to the evolutionary views on the relation between emotion and action readiness. In any case, our study highlights the profound role of auditory emotional processing on action preparation in general.

Overall, we found that the CST excitability significantly increased in response to unpleasant as compared to neutral sounds. This result is consistent with studies reporting overall increased activities in areas related to action representation and motor areas during the presentation of fearful body expressions [11], [12]. Moreover, this result supports the notion that unpleasant stimuli are usually associated with dangerous or painful situations that may lead to a higher action readiness and trigger stronger fight-or-flight responses than positive stimuli [75].

We found that the CST excitability is asymmetrically modulated as a function of the stimulus valence: Unpleasant stimuli caused a significantly higher facilitation in the left hemisphere and pleasant stimuli in the right one. Our findings are consistent with other previous studies that showed larger MEPs elicited by left M1 stimulation during the presentation of negative spoken scenarios [21], the exposure to unpleasant images [14] and fearful facial expressions [17]. The results we obtained following the stimulation of right M1 complement those by Baumert and co-workers [21] who did not find modulation of CST excitability in response to positive scenarios when TMS was applied to left M1. By contrast, we cannot support the findings of Hajcak and co-workers [15] and van Loon and co-workers [18] who found larger MEPs in the left hemisphere while participants observed both pleasant and unpleasant compared to neutral images. Our results are in complete agreement with a study that reported motor facilitation through TMS over left M1 during self-induced sadness, while imagination of happy thoughts induced selectively enhanced right CST excitability [20]. We extended this evidence by showing a hemispheric asymmetry in CST excitability depending on emotional valence of non-verbal sounds, and we controlled for the degree of valence using standard stimuli.

We note that the present results contrast the patterns of lateralization in emotional processing as suggested by valence, motivational, and right hemisphere models. One possible explanation might be related to the specificity of the brain regions investigated to shape the models. As mentioned above, Wager and co-workers’ meta-analysis revealed that distinct hemispheric lateralization appeared when small brain regions were analyzed, which was absent when the gross activity of the entire right vis-à-vis left hemisphere was investigated [55]. That is, lateralization for emotional processing may change from region to region. This was in fact what let us focus on the lateralization of M1. Interestingly, there are more inconsistencies in the literature: Most TMS studies on emotional processing reported contradictory results for both valence and right hemisphere models. In these studies, TMS was applied over the left hemisphere and all reported modulation of MEPs during emotional processing, which is inconsistent with the general right hemisphere hypothesis. To date, only a single TMS study [19] has reported results in favor of right hemisphere, valence and motivational hypotheses by showing greater CST excitability in response to unpleasant pictorial stimuli as compared to pleasant and neutral ones. Previous research has also been inconclusive regarding left hemisphere motor facilitation in response to only negative emotions [14], [16], [17], [20], [21] versus both negative and positive emotions [15], [18]. The latter is in contrast with both the valence and the motivational models. Valence and motivational models have been largely derived from EEG resting state asymmetry with focus on prefrontal cortex, which again may differ substantially from lateralization of M1. As such, we believe that the aforementioned discrepancies reflect different facets of a complex distributed system for processing emotions.

It has also been proposed that the functional asymmetry in motor cortex might have been developed as a product of handedness [76]. For instance, the right hand dominancy is highly correlated with functional and structural lateralization in language processing; 95.5 to 99.67% of the right-handers display language dominance in the left hemisphere [77]. Regarding our results, the selective facilitation of the dominant hand motor cortex (left) in response to unpleasant stimuli might reflect the preference and the usage of more capable hand for fast fight-or-flight responses. This hypothesis might be confirmed by testing left handed subjects. On the other hand, we found that pleasant stimuli resulted in a significantly higher facilitation of motor potentials evoked in the right hemisphere. This result suggests that the neural system mediating this effect might have been developed to avoid the competition between the two hemispheres for controlling the muscles involved in approach or avoidance-related actions. The corpus callosum might play an essential role in the development of such hemispheric asymmetry. A number of studies propose that the corpus callosum provides the pathway through which each hemisphere can inhibit the other in order to predominate a given function to allow for more effective intra-hemispheric processing [78], [79].

Along with the hemispheric asymmetry, we further demonstrated ear differences in terms of provoked activity in the motor cortex. Overall, there was an increase in CST excitability when subjects were listening to unpleasant sounds with the left ear as compared to the right one. This finding adds to prior work on the neuro-modulatory role of the left ear in emotional processing which identified left-ear advantage in recognition of emotional sounds [59]–[64]. In these studies, left-ear advantage was reported for both pleasant-unpleasant stimuli (supporting the right hemisphere hypothesis), whereas we identified a left ear lateralization just in response to unpleasant sounds in terms of overall increase in CST excitability. An interaction between ears, hemispheres and emotional valence conditions was not statistically significant but the increment of CST excitability in response to unpleasant sounds for left M1 stimulation along with the major contribution given by the left ear lets us speculate that unpleasant or threatening auditory stimuli might be processed via a left ipsilateral projection. Indeed, several neuroimaging studies suggested that input from each ear projects to both contralateral and ipsilateral auditory cortex (the contralateral being the dominant one) [80]–[82]. More recently, similar bilateral cortical activations following monaural and binaural auditory stimulation have been reported [83], [84]. This supports the idea that the involvement of different brain areas in processing auditory inputs may depend on the type of information being conveyed rather than just being stronger in contralateral and weaker in its ipsilateral hemisphere.

We did not find significant differences between CST excitability when comparing binaural with monaural stimulation (Figure 2, panel C). As suggested by other experiments conducted by Goycoolea and co-workers [83]–[84], the brain activities that result from a binaural stimulation should not be considered as a mere summation of two monaural stimulations but rather as an ‘integration of information’ for optimal processing; they found higher activation in response to monaural as compared to binaural stimulation of pure tones. We also did not find significant differences between the MEPs elicited during binaural and monaural stimulation of unpleasant sounds. We here conclude that, depending on the type of information conveyed, monaural and binaural stimulation yields different brain activities.

Listening to sounds with the left ear yielded significantly larger MEPs evoked by unpleasant ones as compared to neutral and pleasant sounds. It might be that the left ear is more sensitive to unpleasant sounds and might thus be the primary trigger for fight-or-flight responses.

As every study, also the current one has its limitations, which may put conclusions into perspective. First, auditory evaluations of the participants were realized using a self-hearing test [70]. This choice does not allow for comparing left/right hearing performance individually. Was hearing performance a confounding factor in our study? We cannot answer this but will employ a more detailed audiometric evaluation in future studies. Second, since we focused on response differences as a consequence of emotional valence, we kept the stimuli as natural as possible. By doing this, however, other physical characteristics like the spectral composition of the stimuli might have differed so much that this affected CST excitability. Again, we refer to future studies to investigate CST excitability as a function of different spectral characteristics in more detail. Third, in the present study the auditory stimuli were presented using a supra-aural earphone (Beyerdynamic DT-770), which has a low amount of interaural attenuation and thus a risk of cross-over. The term interaural attenuation refers to the amount of energy reduced or weakened when the sound is transmitted across or trough the skull from one ear to the other and can depend on the earphone transducer type [84]. However, to what extent this mechanical cross-over might have affected the CST excitability remains unclear.

### Conclusions

Our findings reveal a hemispheric specialization as a function of the stimulus valence, which suggests the existence of a lateralized auditory-motor pathway in response to unpleasant emotional sounds but not for pleasant ones. The increment of corticospinal motor excitability in the left primary motor cortex in response to unpleasant sounds along with the major contribution given by the left ear could suggest the presence of a preference for a direct motor-auditory projection for processing threatening auditory stimuli. This system might have been developed to allow for faster fight-or-flight responses to potential dangerous stimuli. However, the neural mechanisms underling this asymmetry remains to be investigated. We believe that future extension of this research approach promise to yield more insight into the nature of such biological preference, which is likely to have been shaped by our evolutionary heritage.

## Supporting Information

Appendix S1 **The numbers of the IADS sounds that were selected as experimental stimuli in the current study.**
(DOCX)
