# Supplementary material for: Excitability of Motor Cortices as a Function of Emotional Sounds
Source: PLoS One. 2013 May 7;8(5):e63060. doi: 10.1371/journal.pone.0063060 (PMC3646985; doi:10.1371/journal.pone.0063060)
Supplement: Appendix S1 — The numbers of the IADS sounds that were selected as experimental stimuli in the current study. (DOCX) [file pone.0063060.s001.docx]

pleasant, 110, 150, 172, 811, 815; neutral, 246, 361, 700, 708, 722; and unpleasant, 116, 293, 626, 714, 719.
